# A dual-track feature fusion model utilizing Group Shuffle Residual DeformNet and swin transformer for the classification of grape leaf diseases

**DOI:** 10.1038/s41598-024-64072-x

**Published:** 2024-06-24

**Authors:** R. Karthik, Gadige Vishnu Vardhan, Shreyansh Khaitan, R. N. R. Harisankar, R. Menaka, Sindhia Lingaswamy, Daehan Won

**Affiliations:** 1 Centre for Cyber Physical Systems, Vellore Institute of Technology, Chennai, 600127 India; 2 School of Computer Science and Engineering, Vellore Institute of Technology, Chennai, 600127 India; 3 Department of Computer Applications, National Institute of Technology, Tiruchirappalli, 620015 India; 4 System Sciences and Industrial Engineering, Binghamton University, Binghamton, 13902 USA

**Keywords:** Grape leaf disease, Deep learning, Dual-track network, Swin transformer, Triplet attention, Computational biology and bioinformatics, Engineering

## Abstract

Grape cultivation is important globally, contributing to the agricultural economy and providing diverse grape-based products. However, the susceptibility of grapes to disease poses a significant threat to yield and quality. Traditional disease identification methods demand expert knowledge, which limits scalability and efficiency. To address these limitations our research aims to design an automated deep learning approach for grape leaf disease detection. This research introduces a novel dual-track network for classifying grape leaf diseases, employing a combination of the Swin Transformer and Group Shuffle Residual DeformNet (GSRDN) tracks. The Swin Transformer track exploits shifted window techniques to construct hierarchical feature maps, enhancing global feature extraction. Simultaneously, the GSRDN track combines Group Shuffle Depthwise Residual block and Deformable Convolution block to extract local features with reduced computational complexity. The features from both tracks are concatenated and processed through Triplet Attention for cross-dimensional interaction. The proposed model achieved an accuracy of 98.6%, the precision, recall, and F1-score are recorded as 98.7%, 98.59%, and 98.64%, respectively as validated on a dataset containing grape leaf disease information from the PlantVillage dataset, demonstrating its potential for efficient grape disease classification.

## Introduction

According to the Food and Agriculture Organization, grapes are cultivated on a significant global land area. The majority of world grape production is allocated to wine, with a notable portion used as fresh fruit and a smaller percentage used as dried fruit. According to the reports of the Indian Council for Agricultural Research, India ranks among the top countries in terms of grape production worldwide^1^. Despite their widespread cultivation and economic importance, grapes are susceptible to various diseases, including isariopsis leaf spot, black measles, and black rot. All of these diseases result in significant losses, affecting overall grape yields. This challenge has broader implications for the global grape industry, emphasizing the importance of addressing and mitigating the impact of diseases on grape cultivation worldwide. Efforts to increase disease resistance and minimize losses are crucial for sustaining and improving the economic viability of grape cultivation globally^2^.

Therefore, identifying grape diseases at the earliest stage has the potential to minimize losses, manage costs, and ultimately enhance the quality of products. For many years, humans have been primarily responsible for identifying plant diseases. Identification and diagnosis are subjective, expensive, time-consuming, and prone to errors. Furthermore, new diseases have the potential to emerge in areas where they have never been detected before and where there is naturally no local knowledge to address them^3^. Thus, identifying an efficient and economical method for identifying crop disease cases is critical. This provides an opportunity for the implementation of Computer-Aided Diagnosis systems in agriculture, utilizing image processing, machine learning, and deep learning techniques to enhance the precision of disease diagnosis^4^. A variety of feature extraction strategies and learning algorithms can be applied to increase classification accuracy. Selecting an optimal image processing method can be challenging and requires technical skills^5^. Promising research on automatic plant disease detection is not without difficulties due to the small size of disease patches on grape leaves. Additionally, environmental conditions are another problem that may influence plant disease detection, making this a challenging task even to date.

Numerous machine learning techniques have been developed for diagnosing plant diseases. Nevertheless, the accuracy of these models heavily relies on human involvement in the extraction of features and complex pre-processing techniques applied to images^6^. Advances in computation have led to the emergence of deep neural networks composed of many artificial neurons stacked in a certain architecture. Similarly, recent research in algal cultivation has leveraged artificial intelligence techniques, such as machine learning and deep learning, for image classification tasks, demonstrating the potential for automated identification and monitoring in agricultural practices^7^. Unlike machine learning methods, these networks can learn features on their own without manually feeding them to the model during training^8^. This increases the number of training parameters, thereby increasing the training time of deep learning models. However, existing research has shown that DL models have higher recognition accuracy than ML models^9^. The main focus of this research revolves around preventing the damage caused by grape leaf diseases, which can lead to economic problems in the agricultural industry. Hence, the objective of this research is to create a system for automatically detecting diseases in grape leaves employing deep learning techniques.

The proposed network for identifying grape leaf diseases addresses several research gaps. First, it uses a feature fusion model to mitigate the substantial loss that can occur when local or global features are independently extracted, thereby enhancing the overall performance of deep learning models in grape leaf disease detection. Second, it introduces deformable convolution with learnable offsets for dynamic sampling, which enhances the model's ability to capture intricate patterns and addresses the limitation of fixed grid patterns in standard convolutional layers. Third, it employs residual blocks with group convolution and channel shuffling to improve the model's generalizability and maintain high accuracy in classification tasks. The key contributions of this research include the adoption of a dual-track network architecture that concurrently considers global and local features, the introduction of deformable convolution with learnable offsets for dynamic sampling, and the utilization of residual blocks with group convolution and channel shuffling.

The following sections of the paper are arranged in the following manner: Section "Related works" delves into the existing works within this domain. Moving forward, Section "Methods" provides a detailed description of the proposed methodology. Moreover, Section "Results and discussion" delves into ablation study on the model and showcases the outcomes of the experimentation. The paper culminates with Section "Conclusion", which offers a conclusive summary.

## Related works

The following section reviews related research focused on grape leaf disease classification. These investigations employ a combination of machine learning and deep learning techniques, and detailed discussions and analyses are presented in the following subsections.

### Machine learning methods

Machine learning models have gained popularity because they focus on detecting diseases in grape leaves by analyzing various datasets. Several ML algorithms have been successfully applied to identify and classify grape leaf diseases. The commonly employed approaches include logistic regression, K-nearest neighbors (KNN), decision trees, Naive Bayes, random forest, and Support Vector Machine (SVM).

Alishba et al. improved grape leaf images through the application of local contrast haze reduction and neighborhood component analysis. Subsequently, they utilized a multiclass SVM model for classifying grape leaf diseases^10^. Javidan et al. applied an image-processing algorithm alongside K-means clustering to distinguish disease symptoms on grape leaves. Subsequently, the application of SVM integrated with Principal Component Analysis facilitated accurate disease classification^11^. Pranjali et al.^12^ utilized an SVM classification technique for detecting and classifying grape leaf diseases. K-means clustering was employed to detect diseased regions, after which texture and color features were extracted to enable accurate disease identification. Haibin et al. introduced a novel method, the Cascade Wavelet Attention Network (CWAN), for accurately identifying grape leaf diseases. CWAN combines a Cascade Wavelet Attention and an autoencoder, effectively addressing issues such as color bleeding, lesion size, and edge smoothness in grape leaf images^13^. Another study on SVM models was presented by Jaisakthi et al. This study introduced an automated system for identifying grapevine diseases utilizing machine learning and image processing techniques. Employing grab-cut segmentation of isolated leaf regions, with disease segments identified via semi-supervised and global thresholding techniques. The extracted features were classified using AdaBoost, SVM, and Random Forest (RF) tree models, which achieved optimal testing accuracy with SVM^14^. Another SVM implemented by Kaur et al.^15^ employed fractional-order Zernike moments and SVM for grape leaf disease recognition. The comparative examination included integer-order Zernike moments and diverse methods for feature selection.

Krithika et al. employed the KNN classification algorithm to identify grape leaf diseases. The approach identifies leaf skeletons from grape images using a segmentation algorithm based on Tangential Direction. Subsequently, histograms and color channel details are extracted from classified grape leaf images, enabling the observation of pixel values to distinguish between diseased and healthy tissues. Features are subsequently extracted and utilized in the KNN classification algorithm for the precise identification of leaf diseases^16^. Kaur et al. proposed a semi-automated system for detecting grape leaf diseases by employing K-Means clustering optimized through Grey Wolf Optimization for segmentation. Hybrid features are extracted using Law’s mask, incorporating Local Binary Pattern, Grey Level Co-occurrence Matrix, and Gabor features. An ensemble classifier is subsequently applied for disease classification^17^. Kirti et al.^18^ utilized a color-based separation technique to separate the features of healthy and diseased parts of leaves. These features are fed to the SVM classifier to identify diseased leaves.

Although ML algorithms play a crucial role in grape leaf disease classification, researchers have started exploring DL methods to achieve better performance. Manual feature extraction is a concern with ML models that require domain expertise. In contrast, DL models automatically extract relevant features, eliminating the need for manual feature extraction.

### Deep learning methods

In recent studies, Deep Learning (DL) methods have emerged as powerful tools for detecting diseases in grape leaves. Researchers have increasingly turned to pre-trained models such as VGG16, AlexNet, EfficientNet, and MobileNet combined with transfer learning techniques to increase the accuracy of grape leaf disease classification.

Sanath et al.^19^ used a modified AlexNet architecture for automatic feature extraction and classification of Grape leaf diseases. Another study implemented a transfer learning approach utilizing the AlexNet architecture for grape variety identification in Douro Region vineyards. Carlos et al.^20^ overcame challenges such as image similarity and seasonal changes and achieved promising results through a pre-processing method and an image warping algorithm. Ananda et al. utilized a CNN, specifically the VGG model. The model was effective in the early detection and classification of diseases in grape leaves, demonstrating enhanced accuracy in distinguishing between diseased and healthy leaves^21^. Miaomiao et al.^22^ proposed a way to detect grape black measles disease by combining the pre-trained ResNet50-based DeepLabV3 + semantic model for segmentation with fuzzy logic. Kirti et al.^23^ utilized the ResNet50 architecture to identify Esca (Black Measles) in GrapeVines using transfer Learning. Alishba et al. introduced an approach for the early detection of grape diseases utilizing a deep learning-based solution. The methodology involves feature extraction through transfer learning on both AlexNet and ResNet101. After feature extraction, the Yager Entropy combined with the Kurtosis technique is applied for feature selection, and fusion is accomplished through a parallel approach^24^.

The research contributed by Hubert et al.^25^ used CNNs and transfer learning to detect two types of grapes in images. It explored various input feature spaces and data augmentation impacts and assessed 11 pre-trained deep learning architectures for grape segmentation. Sood et al.^26^ presented a deep CNN for grape leaf disease identification. During training, Gaussian noise features are integrated to enhance the accuracy. The pre-trained CNN models, namely, VGG16, InceptionV3, ResNet50, and DenseNet121, are used for further classification. Mahmudul et al. employed InceptionResNetV2, InceptionV3, EfficientNetB0, and MobileNetV2 models with modified convolution layers to classify plant images. The evaluation involved adjusting parameters such as batch size and dropout, achieving promising disease classification accuracy rates. The findings demonstrated superiority over conventional handcrafted feature-based approaches and certain other deep learning models^27^.

Haibin et al. introduced a super resolution network designed for the identification of grape leaf diseases. The network utilizes convolution for low-level features and incorporates a dynamically designed learning strategy. This approach effectively balances global and local features, resulting in enhanced accuracy in disease recognition^28^. Zhe et al. proposed a lightweight convolutional neural network for grape disease diagnosis. It emphasizes mobile device compatibility by utilizing ShuffleNet with channel-wise attention via squeeze-and-excitation blocks^29^. Miaomiao et al. introduced UnitedModel, a CNN architecture designed for the automatic identification of common grape diseases^30^. Diana et al. employed convolutional capsule networks to detect grape leaf diseases. The model integrates convolutional layers, streamlining capsule numbers, and expediting dynamic routing^31^. Khalid et al. developed a novel approach employing a lightweight deep CNN for the early identification of plant leaf diseases. This model is designed to reduce the number of parameters for training and the computational complexity compared with traditional transfer learning models. The model captures local spatial texture details in images of plant leaves by integrating deep features alongside handcrafted features based on Local Binary Pattern (LBP)^32^. In another similar work, Rajinder Kumar et al. developed a CNN from scratch for the identification and classification of grape leaf diseases. The TensorFlow tflite format of the model enables real-time disease identification on mobile devices for precision agriculture^33^.

Shtwai et al. used a hybrid deep learning model employing a CNN and Gated Recurrent Unit. It incorporates median Filtering for pre-processing and a Dilated Residual Network for feature extraction. The model optimizes hyperparameters using the Improved Salp Swarm Algorithm for grape leaf disease classification^34^. Furthermore, Chuang et al. introduced a method for grape leaf image enhancement, incorporating a Siamese DWOAM-DRNet architecture for grape disease classification. By utilizing diverse-branch residual modules and a double-factor weight optimization attention mechanism, the Siamese network enhances disease feature extraction and mitigates background complexities^35^. Recent studies have demonstrated that transformer-based networks can achieve faster processing rates and greater accuracy in classifying grape plant diseases. Xiangyu et al. proposed combining Ghost-convolution and Transformer networks for identifying grape leaf diseases. The ghost convolution was used to generate intermediate feature maps and reduce linear computations. The Ghost enlightened Transformer model, namely, GeT, with multi-head self-attention is then employed to extract relevant features. The model is then transfer-learned from ImageNet^36^.

The study by Geetharamani et al. introduced a novel CNN for plant leaf disease classification. The model employs six data augmentation methods, enhancing the performance. The investigation explored variations in training epochs, dropouts, and batch sizes^37^. A comparative analysis indicated that the proposed approach outperformed transfer learning approaches. Yeswanth et al. introduced a novel super-resolution technique known as Residual Skip Network-based Super-Resolution for Leaf Disease Detection. The method involves guided filtering to split low-resolution (LR) images into two components. A single-layer convolutional network and a four-layer two-channel residual skip network are used for feature extraction. These features are concatenated followed by a convolutional layer and decoding block to produce a super-resolution (SR) image. For training, a novel loss function called collaborative loss is introduced, and the enhanced image is fed to the network responsible for early disease detection^38^.

## Methods

The following section starts with a comprehensive overview of the dataset employed in the research. This section is followed by an explanation of the environmental setup, hyperparameter tuning, and a detailed explanation of the proposed network.

### Dataset description

This research uses a grape dataset extracted from the PlantVillage dataset. The grape dataset consists of 3 disease classes, namely, leaf blight, black measles, black rot, and one healthy class. To increase the number of healthy leaf images, the dataset was augmented using six different data augmentation techniques^39^. The classwise statistics of the images before and after data augmentation are shown in Table 1.

**Table 1:** Classwise statistics of the dataset.

Class name	Before data augmentation			After data augmentation		
	Training set	Validation set	Test set	Training set	Validation set	Test set
Black Rot	708	236	236	708	236	236
Esca	829	276	278	829	276	278
Leaf Blight	645	215	216	645	215	216
Healthy	253	84	86	600	200	200
Total	2435	811	816	2782	927	930

### Environment setup

The experiments with the proposed network were carried out in Google Colab using a T4 GPU and PyTorch as the framework. The training environment consists of 16 GB of GPU RAM, 2v CPUs with 13 GB of RAM, and 64 GB of disc space. The Adam optimizer with the cross-entropy loss function achieved better accuracies on the grape dataset.

### Hyperparameter tuning

Hyperparameter tuning plays an essential role in improving model performance. The model hyperparameters are adjusted to increase the model's generalization capability. The dropout rate, learning rate, weight decay, and optimizer are the hyperparameters tweaked in the proposed network. This research involved analyzing the model with a wide range of values for each hyperparameter. Weight decay, Learning rate, Dropout, and optimizer are the hyperparameters selected for this experiment. The optimal hyperparameters are selected using the Grid Search Algorithm. The range of experimented hyperparameters are mentioned in Table 2. The dropout layer and weight decay play important roles in preventing the model from overfitting, thus producing generalized outputs. The dropout layer reduces the codependency among the neurons, leading to better generalization on unseen images. Following a series of experiments, the Adam optimizer with a learning rate of 0.001, a weight decay of 0.0002, and a dropout of 0.3 outperformed the results achieved with other hyperparameters on the final model.

**Table 2:** Hyperparameter tuning.

Parameter	Search space	Optimal value
Weight decay	[0,1e–2, 1e–3, 1e–4, 2e–4, 5e–4]	2e–4
Learning rate	[1e–2, 1e–3, 2e–3, 1e–4]	1e–2
Dropout	[3e–1,4e–1,5e–1]	3e–1
Optimizer	SGD, ADAM	ADAM

### Proposed network

**Figure 1:**
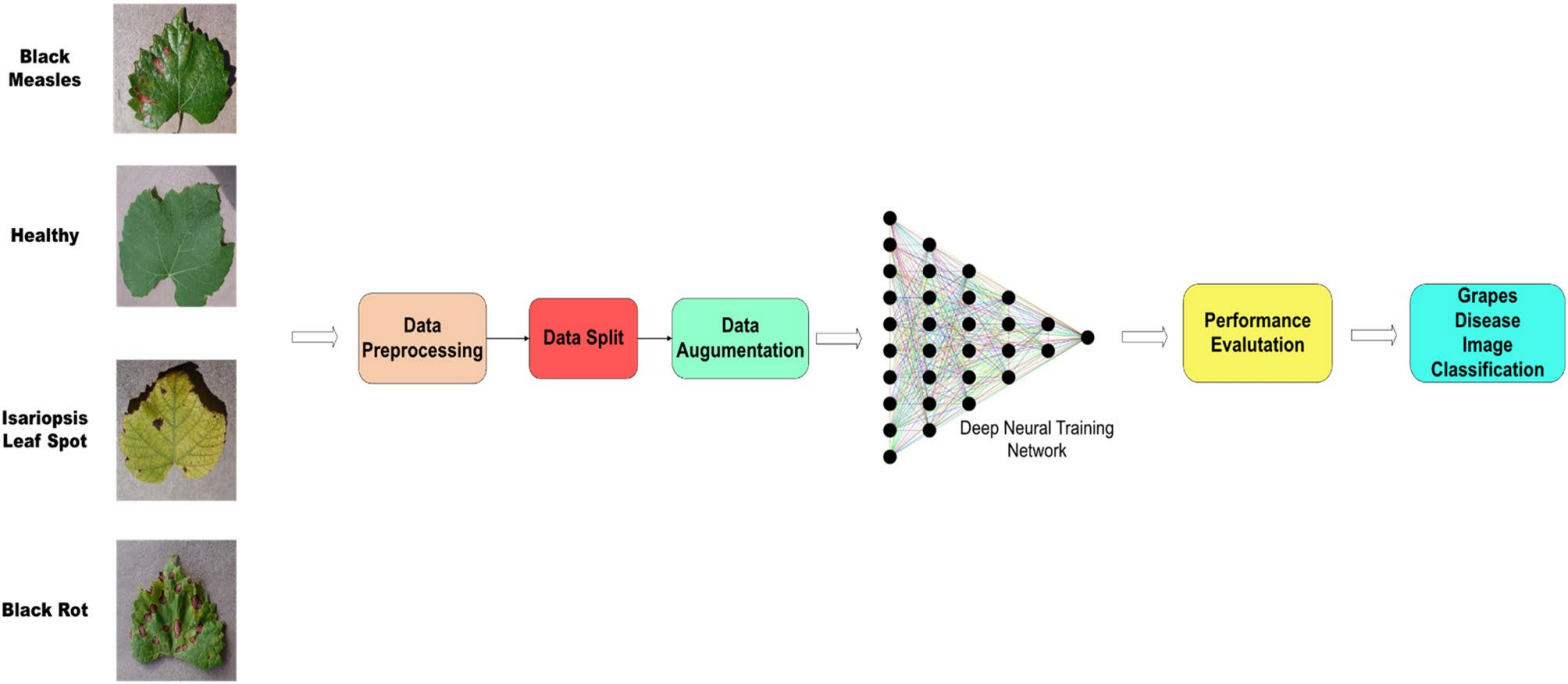
Schematic overview of the proposed approach.

The proposed methodology involves a dual-track approach, with one track adopting the Swin Transformer model and the other track incorporating a novel CNN track. The Swin transformer is used to extract global features, and the GSRDN, a proposed CNN track, extracts local features from the input image. The fundamental concept underlying the presented network is the integration of the Swin Transformer architecture with convolutional neural networks (CNNs) to enhance feature acquisition and improve accuracy. The features extracted from both tracks are combined to train a robust model. Figure 1. shows the process flow of the proposed network.

**Figure 2:**
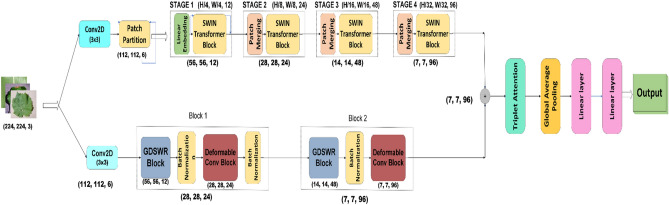
Modular overview of the proposed network.

The swin transformer track consists of a series of Swin transformer blocks packed with patch partition, linear embedding, and patch merging layers. The main purpose of using the Swin Transformer is to construct hierarchical feature maps via the shifted window technique, which can boost the overall model performance via effective feature aggregation. The GSRDN track is a combination of two blocks: 1) a Group Shuffle DepthWise Residual (GSDWR) block that includes Group convolution, Channel Shuffle, and DepthWise separable convolution arranged linearly along with a residual network and 2) a DC block that is an accumulation of Deformable Convolution and Depthwise Separable convolution. Batch Normalization (BN) is used between the blocks to stabilize the network’s learning process. The features from both tracks are concatenated horizontally. Triplet attention (TA) is used for weighing the concatenated features, followed by Global Average Pooling (GAP) for feature reduction. The final classification layer utilizes cross-entropy loss and softmax activation to categorize the input data. A detailed outline of the proposed network is shown in Fig. 2.

#### Swin transformer track

**Figure 3:**
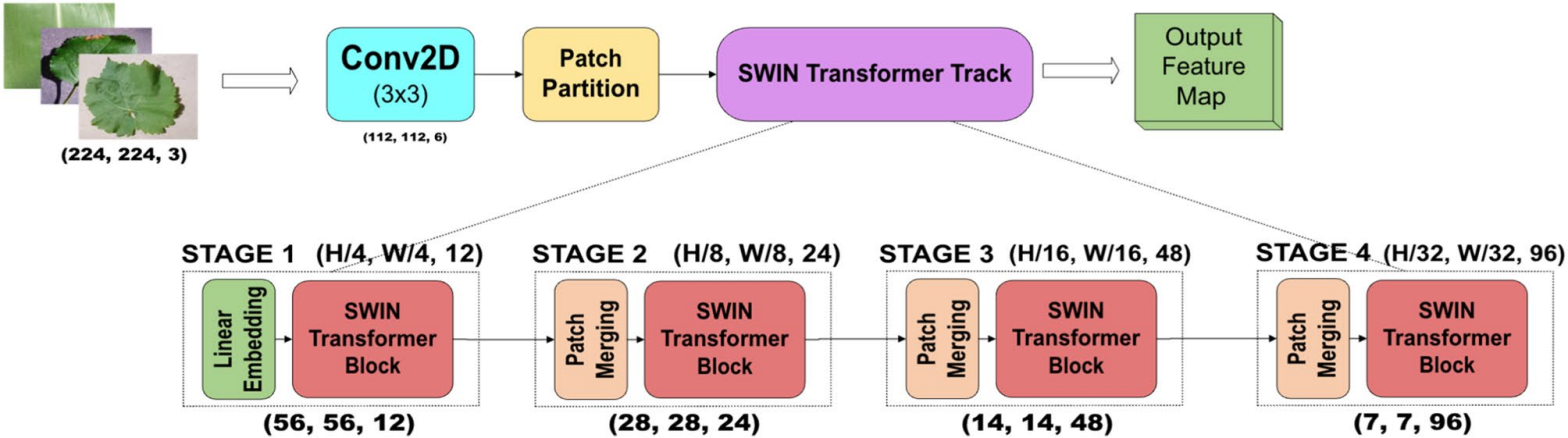
Schematic overview of the Swin transformer track.

A detailed description of the Swin Transformer track is presented in this section. Hierarchical feature maps are built using the shifted Windows technique because of their linear computational complexity. A detailed block diagram of the Swin Transformer is shown in Fig. 3. A four-stage Swin Transformer track is used in the proposed network. The input image is resized before feeding it to the swin transformer track. The processed image is directed through different layers and modules in the track to produce the output feature map.

An input image with dimensions of 224 × 224 × 3 is processed through a standard 2 × 2 2D convolution layer to extract relevant features. The produced feature map is split into non-overlapping patches with a patch size of H × W by a patch partition module. It is followed by four Swin Transformer blocks with self-attention computations employed with linear embedding in the first block and patch merging in later blocks. Each block comprises multihead self-attention, layer normalization, and a 2-layer Multi-Layer Perceptron (MLP) layer with GELU nonlinearity in between. For each swin transformer block, both window Multi-head self-attention (W-MSA) and Shifted Window self-attention (SW-MSA) are used to efficiently capture the global features and long-range dependencies^40^. The number of patches is reduced by patch merging layers to produce a hierarchical representation. The features of each set of adjacent 2 × 2 patches are combined using patch merging layers followed by a linear layer employed on the concatenated features. This downsamples the output feature maps by a factor of 2, making the dimension of the final output feature map H/32 × W/32 × 8C with 96 channels. The feature maps obtained are combined with the output of the GSRDN track for further training.

#### Group Shuffle Residual DeformNet track

**Figure 4:**
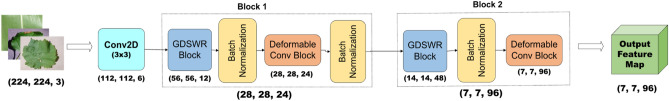
Architectural overview of the GSRDN block.

This section provides a detailed description of the proposed GSRDN track, as shown in Fig. 4. The track comprises two main blocks: Group Shuffle Depth-Wise Residual (GSDWR) block and Deformable Convolution (DC) block, which are grouped sequentially. An input dimension of 224 × 224 × 3 is fed to the network. After every block, the number of channels is doubled, and the resulting feature map dimension is reduced by half. The final output of the track is a feature map with 96 channels. These features are combined with the features from the Swin Transformer track for further processing.

**Figure 5:**
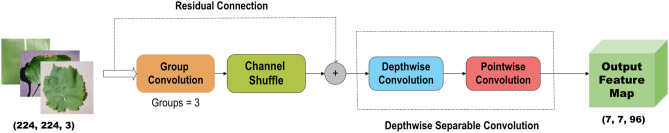
Architectural overview of the GSDWR block.

**Figure 6:**
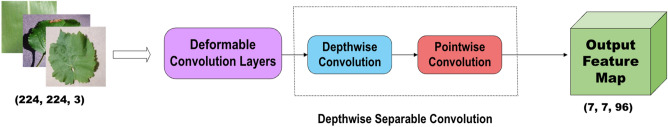
Schematic representation of the DC block.

As illustrated in Fig. 5, the GSRDN track comprises a pair of GSDWR blocks followed by a DC block, with batch normalization layers between the blocks to facilitate faster and more stable network training. The GSDWR block is a combination of Group convolution and channel shuffling packed in a residual network followed by a Depth-Wise separable convolution layer. Group convolution involves dividing channels into groups and applying convolution to each block separately, reducing computational complexity. It is followed by a channel shuffling module that enables cross-group information flow and boosts classification scores by rearranging the channel dimensions^41^. A residual connection is established between the input of GSDWR and the channel shuffling module, which enables the model to learn from both low- and high-level features in the image. The Residual network thwarts the performance degradation problem, thereby increasing model performance ^42^. Finally, Fig. 6 depicts the Depth-Wise Separable Convolution, which is a combination of depthwise convolution and pointwise convolution, making the model computationally cheaper and requiring fewer parameters to train^43^. This reduction in the number of parameters improves the model's efficiency, making it ideal for situations with limited resources or when processing large datasets.

The DC block consists of a deformable convolution layer followed by DepthWise Separable Convolution. The Deformable convolution was introduced in the network to improve the modeling of spatial relationships and adapt to variations in disease shape and positions^44^. This is accomplished by introducing 2D offsets to the regular grid sampling locations, which are learned from the preceding feature maps. This leads to more effective feature learning, as the network can emphasize discriminative image regions and suppress noise or irrelevant parts in the image. A DepthWise Separable Convolution is used as the final layer in the DC block to minimize the number of trainable parameters, thereby decreasing the computational cost of the block.

#### Triplet attention block

**Figure 7:**
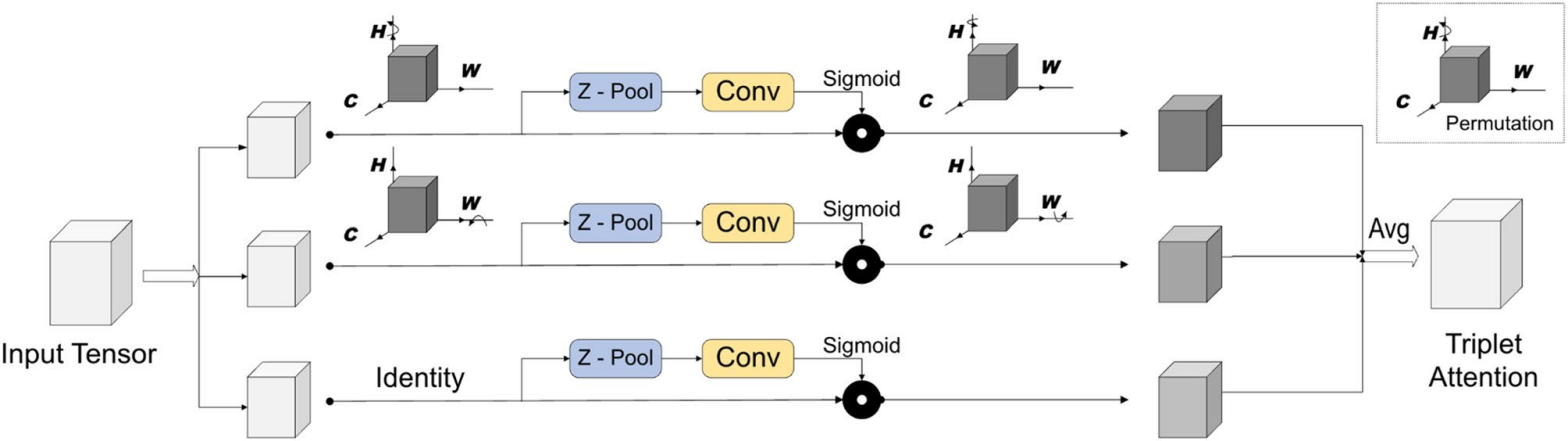
Architectural overview of Triplet Attention block.

This section provides a detailed description of the Triplet Attention (TA) block. Triplet attention, as the name implies, is a three-branch module that calculates attention weights by capturing cross-dimensional interactions between channels. A detailed schematic of the Triplet Attention block is presented in Fig. 7. The 1st and 2nd branches capture the cross-dimensional interaction between the channel dimension and either the spatial dimension H or W. The final branch is tasked with capturing the relation between spatial dependencies H and W. Every branch in the TA comprises a z-pool layer and a convolution layer. The z-pool layer helps to reduce computational complexity by reducing the number of channels in the input tensor to two^45^.

The output tensor obtained by concatenating feature maps from the GSRDN and Swin Transformer tracks is fed to three branches in parallel. In the topmost branch, the input tensor is rotated 90 degrees counterclockwise along the spatial dimension ‘H’ to build interactions between height and channel dimension. The rotated tensor is fed into a z-pool layer, a convolutional layer, and a batch normalization layer. The generated attention weights are fed to a sigmoid activation layer and rotated by 90 degrees along the H axis in the clockwise direction to retain the original input shape. Similarly, the middle branch is rotated along the W axis. For the last layer, the input is passed through the z-pool and convolutional layers without any rotation. The refined tensors from the three branches are concatenated via simple averaging to obtain the final output tensor. Overall, the TA network preserves the relationship between spatial and channel dimensions, thereby increasing model performance.

#### Classification

Global Average Pooling is applied to the feature maps generated from the TA block. It downsamples the entire feature map into a one-dimensional vector, thereby decreasing the number of model parameters in the network. The final block consists of two neural network layers for the multiclass classification of grape diseases. The initial layer comprises 512 neurons with ReLU activation, and the final layer has 4 neurons with SoftMax activation and a cross-entropy loss function for better classification results.

### Plant guideline statement

This study solely utilized digital images, ensuring no physical involvement with endangered plant species. The research adhered to the principles outlined in the Declaration of IUCN Policy on Research Involving Endangered Species and the Convention on Trade in Endangered Species of Wild Fauna and Flora.

## Results and discussion

The following section starts with ablation studies that involve breaking down the model into small components. The following section elaborates on the performance analysis of the overall model, concluding with a discussion of the limitations of the proposed model and the scope for future work.

### Ablation studies

The ablation study performed on the proposed model is discussed in this section. The primary objective aims to construct an efficient model by testing different combinations of layers and blocks to increase its performance on the grape dataset. Different metrics such as accuracy, precision, recall, and F1 score, are employed to evaluate the performance of the proposed model. Additionally, the ROC curve and confusion matrix were also analyzed. The incremental performance of the model is assessed by adding blocks to the network.

#### Analysis of the swin transformer track

**Figure 8:**
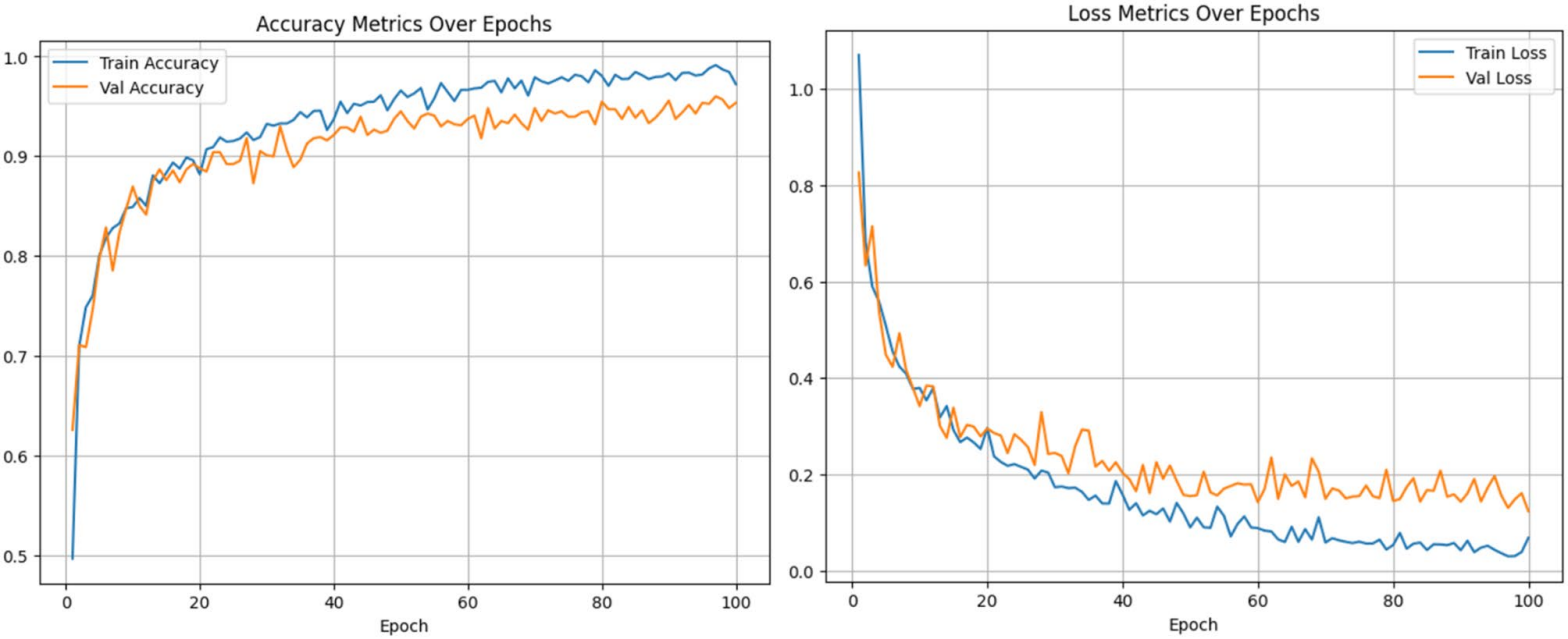
Accuracy and loss plots for the Swin Transformer track.

**Figure 9:**
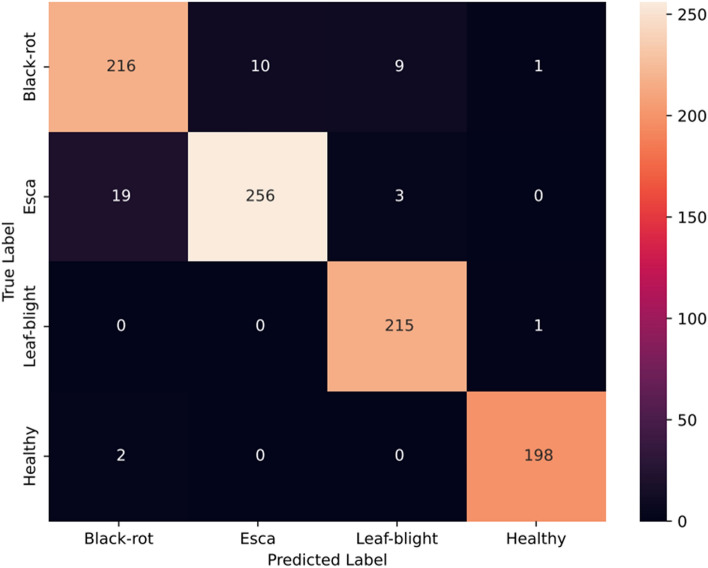
Confusion matrix for the Swin Transformer track.

The Swin transformer track comprises a series of patch partitions, linear embedding, patch merging, and Swin blocks arranged sequentially. The self-attention capability of the Swin transformer extracts features that comprise both local and global information. This track is trained for 100 epochs using an Adam optimizer with a learning rate of 0.001 and a cross-entropy loss function to minimize the training loss. The accuracy and loss after each epoch of training are depicted in Fig. 8. An accuracy of 95.27% was achieved with the track. The other metrics obtained, such as precision, recall, and F1-score are 95.37%, 95.64%, and 95.48% respectively. Figure 9 depicts the confusion matrix obtained for the Swin transformer track.

#### Analysis of the CNN track without deformable convolution

**Figure 10:**
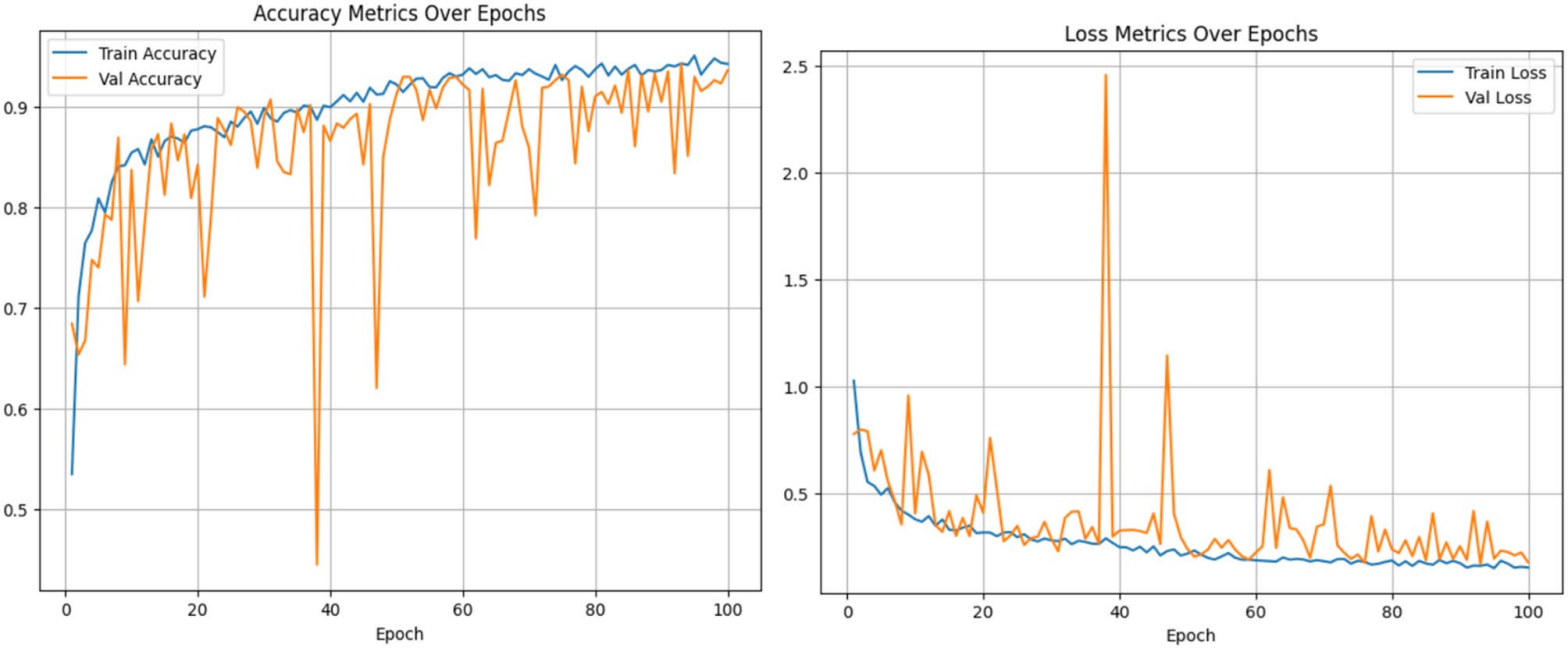
Accuracy and loss plots for the proposed CNN track without Deformable convolution.

**Figure 11:**
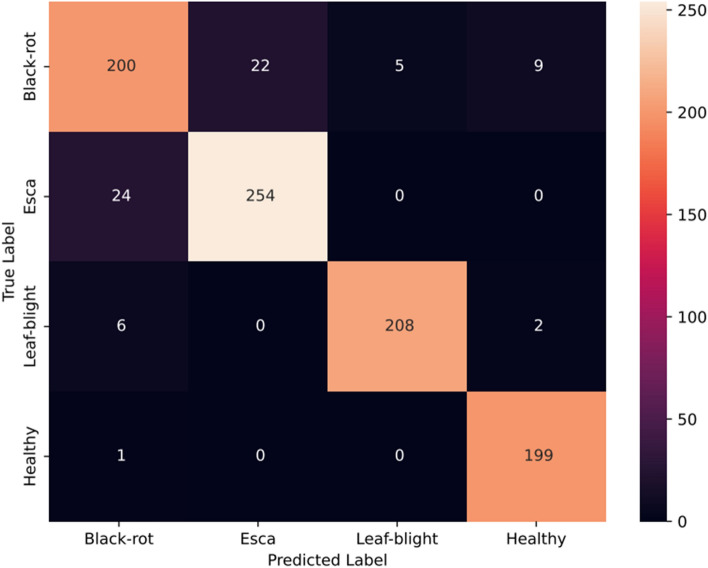
Confusion matrix for the CNN track without Deformable convolution blocks.

The primary objective of the CNN track is to extract local features efficiently. We analyze the performance of GSDWR blocks in this section. The GSDWR block is designed to enhance the relevant feature extraction capability by enabling cross-channel information flow in a computationally cheaper way. The group convolution and Depth-wise separable convolution boost the performance of the track in addition to decreasing the memory utilization. The Adam optimizer is used to train the model for 100 epochs with a cross-entropy loss function. A learning rate and weight decay of 0.001 and 0.01 respectively, achieved the best performance with the blocks used. The accuracy, precision, recall, and F1-score obtained are 94.41, 94.82, 94.48, and 94.51 respectively. The loss and accuracy plots obtained during training are illustrated in Fig. 10. Figure 11 depicts the confusion matrix obtained for the CNN track without the Deformable convolution block.

#### Analysis of the proposed Group Shuffle Residual DeformNet track

**Figure 12:**
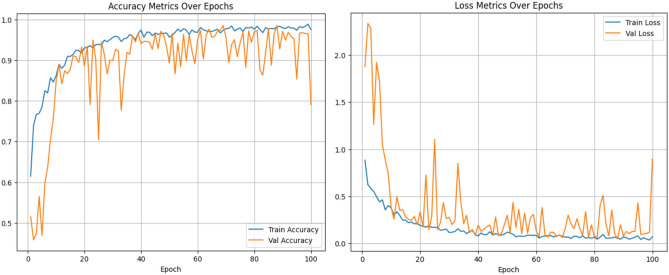
Accuracy and loss plots for the GSRDN Track.

**Figure 13:**
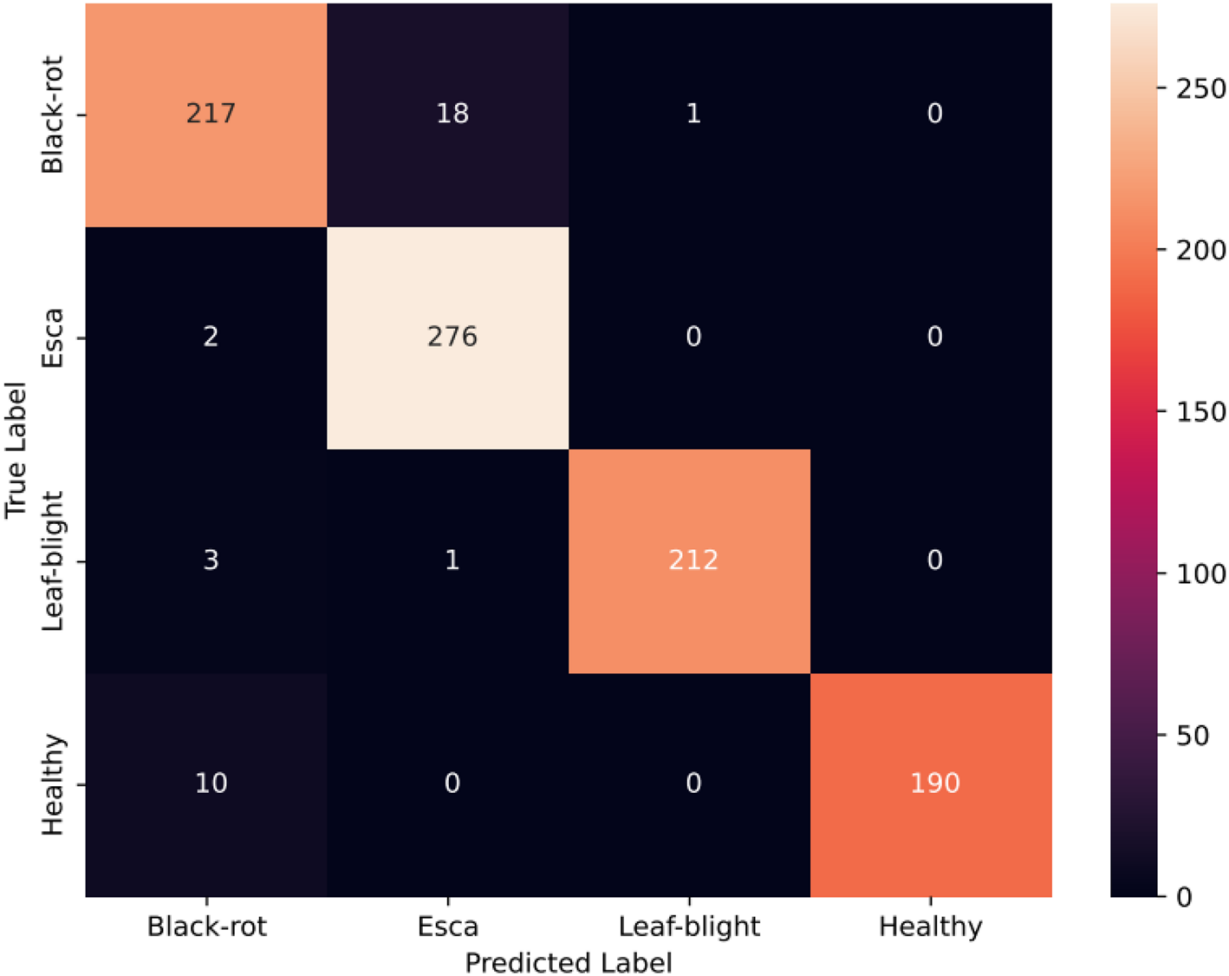
Confusion matrix for the GSDWR track.

The proposed CNN track with GSDWR and a DC block with deformable convolution is analyzed in this section. The receptive field of the deformable convolutional layer is adaptively adjusted for different spatial locations within the image. This allows the model to focus on relevant image regions while ignoring the less informative regions. Subsequently, the Depth-wise separable layer extracts relevant features from the output of deformable convolution in a memory-efficient manner, increasing model efficiency. These features are transferred to a two-layered feed-forward network for classification. The Adam optimizer is used to train the model for 100 epochs with a cross-entropy loss function. A learning rate of 0.001 and weight decay of 0.0005 achieved the best performance with the blocks used. The accuracy, precision, recall, and F1-score obtained are 96.02, 96.5, 95.85, and 96.13, respectively. The accuracy and loss plots obtained during training are shown in Fig. 12. Figure 13 depicts the confusion matrix obtained for the proposed Group Shuffle Residual DeformNet track.

#### Analysis of the overall network without triplet attention

**Figure 14:**
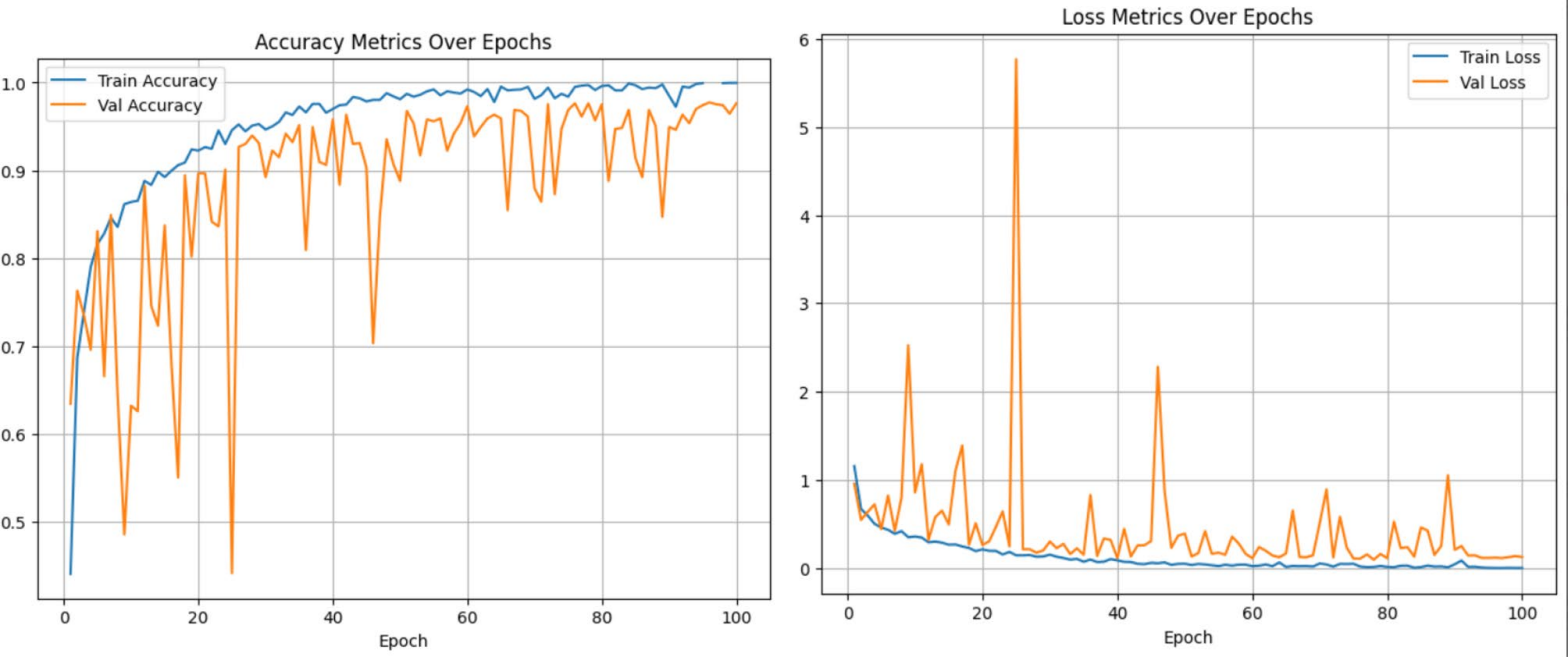
Accuracy and loss plots for the overall network without Triplet Attention.

**Figure 15:**
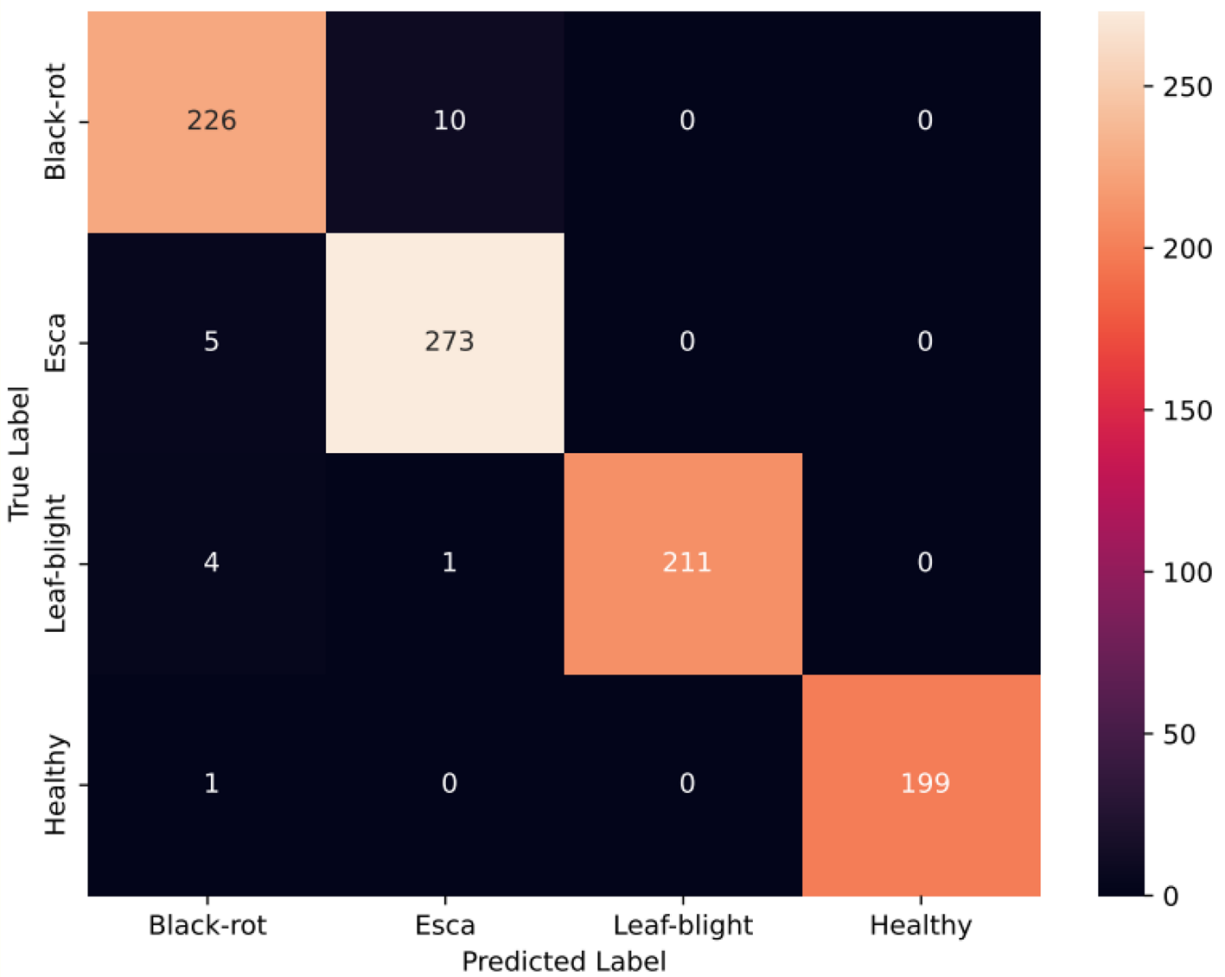
Confusion matrix for the overall network without Triplet Attention.

The output feature maps from both tracks are concatenated to obtain a single feature map. The concatenated feature map is capable of understanding both the local and global content in the given input image. In this section, we evaluate the overall network without triplet attention. An Adam optimizer is used for training this combined model for 100 epochs with a cross-entropy loss function. A weight decay of 0.0001 and a learning rate of 0.001 achieved the best performance with the blocks used. The accuracy, precision, recall, and F1-score obtained are 98.17, 98.32, 98.21, and 98.27, respectively. Combining the features from both tracks helped boost the model's performance. The cross-dimensional interaction capability of triplet attention will significantly contribute to the overall effectiveness of the network. Figure 14 shows the accuracy and loss after each epoch. Figure 15 depicts the confusion matrix obtained for the overall network without Triplet Attention.

#### Analysis of the proposed network

**Figure 16:**
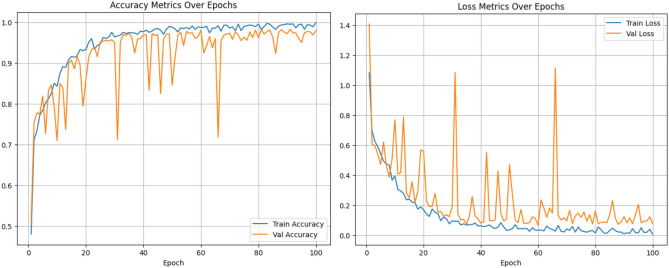
Accuracy and loss plots for the proposed network.

**Figure 17:**
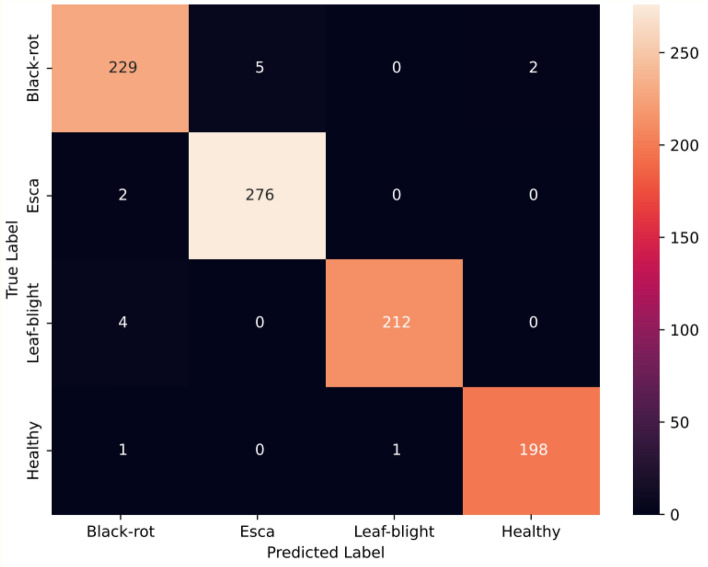
Confusion matrix for the proposed network.

**Figure 18:**
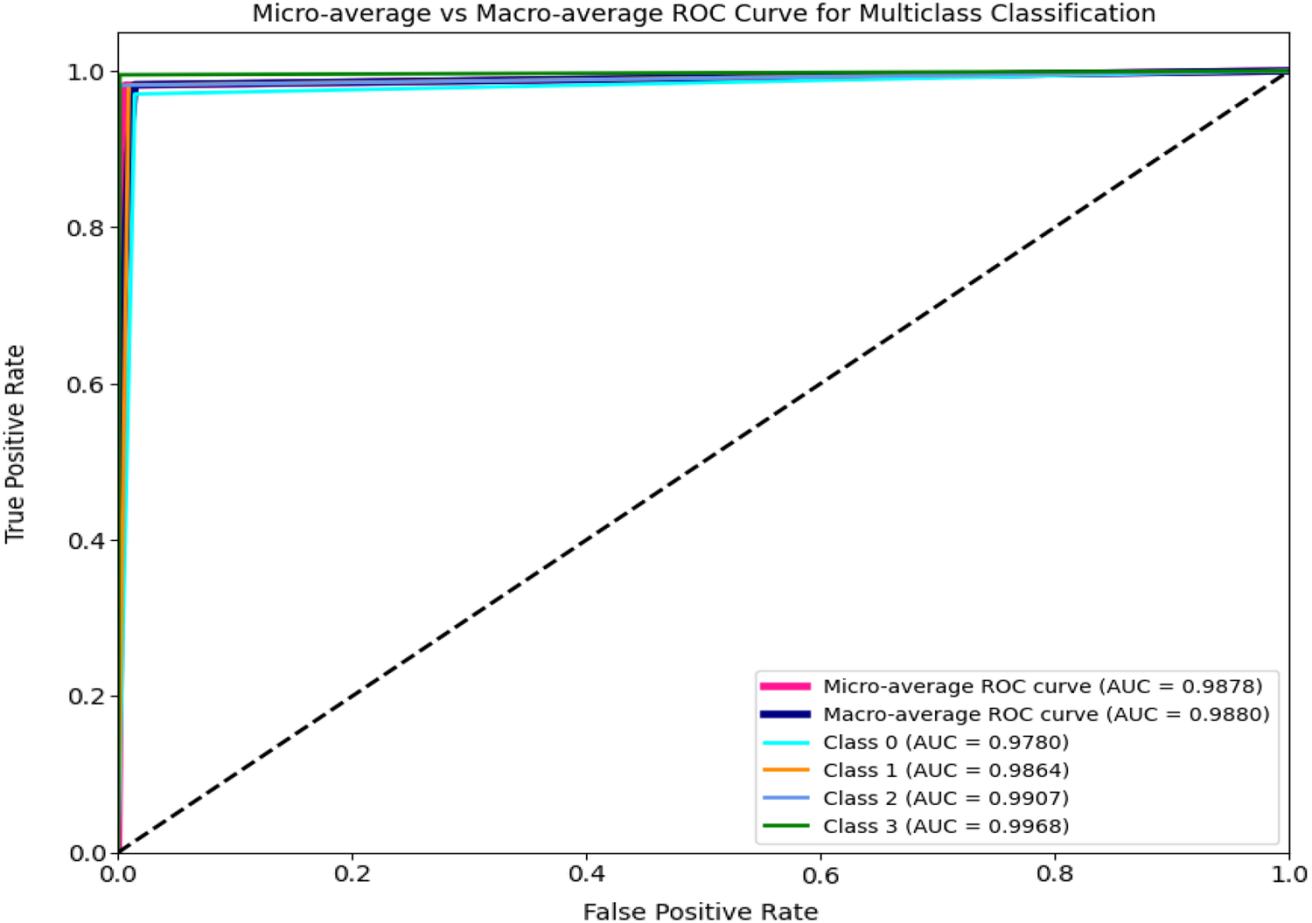
ROC curve for the proposed network.

This section analyses the overall performance of the proposed network with triplet attention. Triplet Attention preserves the interrelation between the spatial and channel dimensions, adding value to the final network. The Adam optimizer is used to train this network for 100 epochs along with the cross-entropy loss function. The accuracy increased to 98.60% with the addition of triplet attention. Figure 16 shows the training phase observations. The precision, recall, and F1-score are recorded as 98.7%, 98.59%, and 98.64%, respectively. Figures 17 and 18 depict the confusion matrix and the ROC curve obtained for the final network, respectively. The obtained ROC curve’s proximity to the top-left corner suggests nearly optimal performance. This positioning indicates a low false positive rate and a high true positive rate, demonstrating the model’s accuracy in predicting the positive class efficiently. Additionally, Table 3 provides a summary of the conducted ablation studies and the observed metrics.

**Table 3:** Overview of the ablation studies.

Model architecture	Accuracy (in %)	Precision (in %)	Recall (in %)	F1 Score (in %)
Swin transformer	95.27	95.37	95.64	95.48
Proposed CNN without novel blocks	93.12	93.2	93.48	93.24
Proposed CNN without DC	94.41	94.82	94.48	94.51
Group Shuffle Residual DeformNet	96.02	96.5	95.85	96.13
Proposed network without triplet attention	98.17	98.32	98.21	98.27
Proposed network	98.6	98.7	98.59	98.64

### Performance analysis

This research proposes a novel approach to efficiently classify grape leaf diseases using the proposed network. The following section compares the results of the proposed network with the existing works reported in related works. A comparison was performed with existing research works that used grape leaf disease data from the PlantVillage dataset, ensuring that the complexity of the images used was similar to that of the images in Table 4. The proposed model outperformed many existing works on the dataset, achieving an improved performance of 98.60%.

**Table 4:** Performance comparison of the proposed work with existing works.

S no	Source	Methods	Accuracy (in %)
1	Kaur et al.^17^	ANN, SVM, KNN	95.69
2	Geetharamani et al.^37^	9-layer deep convolutional neural network	96.46
3	Paymode et al.^21^	VGG16	98.40
4	Ji et al.^22^	ResNet-50 and Inception V3	98.57
5	Proposed network	Swin Transformer + GSRDN	98.60

The dataset was trained with five state-of-the-art pretrained models to evaluate the performance of the proposed network. VGG16, AlexNet, MobileNetV2, EfficientNet-B0, and DenseNet121 are the pretrained models used. The performance analysis of the pretrained models is shown in Table 5. The results showed that the maximum accuracy was obtained by DenseNet101 (98.39%), and the lowest accuracy was obtained by mobileNet_v2 (87.31%). The highest recorded metrics are precision of 98.5%, recall of 98.45%, and F1-score of 98.46%. However, the proposed model was able to achieve better performance than the pretrained model with fewer parameters. We used Google Colab with T4 GPU for all the experiments. It took us 110 min to complete the model training for 100 epochs, with approximately 1 min and 10 s for every epoch. To conclude, the proposed model achieved higher accuracy than many of the previous works in a computationally efficient manner. Future works can explore various other vision transformer models combined with the power of CNNs for better image classification tasks.

**Table 5:** Comparison with state-of-the-art architectures.

Model	Number of parameters	Accuracy (in %)	Precision (in %)	Recall (in %)	F1-Score (in %)	AUC (in %)
VGG-16	134,276,932	93.98	94.35	93.98	94.03	96.32
AlexNet	57,020,228	95.59	95.59	95.59	95.57	97.07
MobileNet-v2	2,228,996	87.31	87.38	87.31	87.27	93.28
EfficientNet-B0	4,012,672	96.24	96.47	96.24	96.25	96.22
DenseNet-201	6,957,956	98.39	98.39	98.39	98.38	98.33
Proposed network	1,528,112	98.6	98.7	98.59	98.64	98.49

## Conclusion

Grape cultivation is vital to the global agricultural economy, yielding diverse grape-based products. However, the susceptibility of grapevines to diseases poses a significant threat to yield and quality. Traditional disease identification methods, which rely on expert knowledge, struggle with scalability and efficiency. To address the challenges of grape leaf disease classification, this research introduces a novel dual-track deep-learning network. This approach integrates global and local feature extraction for enhanced accuracy and efficiency. The proposed model employs the Swin Transformer for global feature learning and leverages a novel Group Shuffle Residual DeformNet (GSRDN) architecture for local feature extraction. The fusion of features from both tracks and the application of the Triplet Attention block for cross-dimensional interactions contribute to the model's robust performance. The model achieves state-of-the-art results on the PlantVillage dataset, with an accuracy of 98.6%, precision of 98.7%, recall of 98.59%, and F1-score of 98.64%.The proposed network currently utilizes images obtained under restricted conditions. A promising future direction would be to extract grape leaf images through aerial photography methods like drones and apply real-time disease classification and image detection techniques to the images for faster identification of diseased plants.

## Data Availability

The datasets generated and/or analyzed during the current study are available in the Plant Village repository. Link: https://data.mendeley.com/datasets/tywbtsjrjv/1.
